# A new potential secretion pathway for recombinant proteins in *Bacillus subtilis*

**DOI:** 10.1186/s12934-015-0374-6

**Published:** 2015-11-10

**Authors:** Guangqiang Wang, Yongjun Xia, Zhennan Gu, Hao Zhang, Yong Q. Chen, Haiqin Chen, Lianzhong Ai, Wei Chen

**Affiliations:** School of Medical Instrument and Food Engineering, University of Shanghai for Science and Technology, Shanghai, 200093 People’s Republic of China; State Key Laboratory of Food Science and Technology, School of Food Science and Technology, Jiangnan University, Wuxi, 214122 People’s Republic of China; Synergetic Innovation Center of Food Safety and Nutrition, Wuxi, 214122 Jiangsu People’s Republic of China

**Keywords:** Non-classical protein secretion pathway, Recombinant protein secretion, Secretory expression, *Bacillus subtilis*

## Abstract

**Background:**

Secretion of cytoplasmic expressed proteins into growth media has significant advantages. Due to the lack of an outer membrane, *Bacillus subtilis* is considered as a desirable ‘cell factory’ for the secretion of recombinant proteins. However, bottlenecks in the classical pathway for the secretion of recombinant proteins limit its use on a wide scale. In this study, we attempted to use four typical non-classically secreted proteins as signals to export three recombinant model proteins to the culture medium.

**Results:**

All four non-classically secreted proteins can direct the export of the intrinsically disordered nucleoskeletal-like protein (Nsp). Two of them can guide the secretion of alkaline phosphatase (PhoA). One can lead the secretion of the thermostable β-galactosidase BgaB, which cannot be secreted with the aid of typical Sec-dependent signal peptides.

**Conclusion:**

Our results show that the non-classically secreted proteins lead the recombinant proteins to the culture medium, and thus non-classical protein secretion pathways can be exploited as a novel secretion pathway for recombinant proteins.

**Electronic supplementary material:**

The online version of this article (doi:10.1186/s12934-015-0374-6) contains supplementary material, which is available to authorized users.

## Background

The downstream processing of proteins is greatly facilitated if the protein of interest is secreted into the culture medium, because recovery of these proteins minimises contamination from host proteins. Since the emergence of recombinant DNA technology, numerous efforts have been made to exploit secretion systems to export recombinant proteins into the culture medium [1–4]. Proteins are more easily exported into the extracellular medium of Gram-positive bacteria than Gram-negative bacteria, because these proteins need only cross the cytoplasmic membrane to reach the extracellular environment. Thus, Gram-positive bacteria particularly *Bacillus subtilis* has been extensively tested for the production of recombinant proteins [5–9]. The advantages of using *B. subtilis* over other bacteria make it one of the most promising hosts for the production of heterologous secretory proteins. These advantages include simplified downstream processing, powerful secretion ability, amenableness to genetic modification, mature fermentation technology, ‘generally recognised as safe’ (GRAS) status, and non-biased codon usage [6].

*Bacillus subtilis* naturally secretes large amounts of proteins directly into the culture medium. Based on predictions from signal peptides, *B. subtilis* has the potential to export approximately 300 proteins. About 50 % of the 113 identified proteins in the extracellular proteome of *B. subtilis* contain typical signal peptides [7]. Most of them are transported via the major Sec pathway. Only two specific substrates are exported via the Tat pathway [8]. A great number of heterologous proteins have been exported by *B. subtilis*, the overwhelming majority of them through the major Sec pathway [9]. However, there are many bottlenecks during Sec-dependent protein secretion, and the secretion of heterologous proteins is frequently inefficient [10]. In addition, cytoplasmic proteins usually cannot be exported across the cytoplasmic membrane into the growth medium with the aid of typical signal peptides [9]. These factors limit the use of *B. subtilis* as a cell factory on a wide scale.

Of the 113 identified proteins in the extracellular proteome of *B. subtilis*, 50 % were predicted not to be released into the extracellular milieu due to cell-envelope retention signals to the membrane or the cell wall, or because of the absence of signal peptides. About 15 % of the 113 identified proteins lack the typical signal peptide and can be considered primarily cytoplasmic proteins [7]. The presence of cytoplasmic proteins in the medium may be attributable to cell lysis. However, many primarily cytoplasmic proteins of several bacterial species have been detected in the extracellular medium [11, 12]. Yang et al. confirmed that the secretion of these proteins without signal peptides in *B. subtilis* is not a consequence of cell lysis and is a general phenomenon [13]. Such proteins are described as ‘non-classically secreted proteins’ because none of the classical secretion systems are involved in their secretion, and this type of secretion is termed ‘non-classical protein secretion’ [14]. Recently, scientists have paid more attention to the mechanisms of non-classical protein secretion, and at least six different export pathways have been identified in various bacteria [15]. Kouwen et al. found that the large conductance mechanosensitive channel protein MscL can prevent the release of specific cytoplasmic proteins during hypo-osmotic shock in *B. subtilis*, and that specific protein release could not be attributed to cell death or lysis [16]. These findings imply that there is an unidentified pathway for the selective release of cytoplasmic proteins in *B. subtilis*. Owing to the in-depth research, the mechanisms of non-classical secretion are becoming clear. However, no study has so far focused on using non-classically secreted proteins to export recombinant proteins into the culture medium.

In this study, we investigated the ability of four typical non-classically secreted proteins to act as signals to export three recombinant proteins to the culture medium. The naturally unsecretory and intrinsically disordered domain of nucleoskeletal-like protein (Nsp) is an ideal tool to study protein secretion [17]. When fused with Nsp all four of these non-classically secreted proteins were able to direct its export. Because alkaline phosphatase (PhoA) contains intramolecular disulfide bonds, PhoA has the activity only when secreted. Moreover, PhoA retains its activity even with an amino-terminal extension. So it has been widely used as a reporter protein in secretion studies [18]. When the four non-classically secreted proteins were fused with PhoA, two of them were able to direct its export. One of the four was able to direct the secretion of the thermostable reporter protein β-galactosidase (BgaB), which cannot be secreted with the aid of typical Sec-dependent signal peptides. These results show that non-classically secreted proteins are sufficient to export recombinant proteins to the culture medium, and non-classical protein secretion pathways can be exploited as a novel secretion pathway for recombinant proteins.

## Results

### Secretion of unstructured Nsp with non-classically secreted proteins

Seventeen non-classically secreted proteins have been listed in an excellent review on protein secretion in *B. subtilis* [7]. These proteins are typical cytoplasmic proteins without classical signal peptides. Notably, glyceraldehyde 3-phosphate dehydrogenase (GapA), superoxide dismutase (SodA), unknown protein which is similar to plant metabolite (YvgN) and pyruvate dehydrogenase E3 subunit (PdhD) have been detected outside the cells of *Bacillus licheniformis* [19], *Listeria innocua* [20], *Staphylococcus aureus* [21, 22] and *Lactococcus lactis* [23] by different research groups. These four proteins are the common non-classically secreted proteins in different bacteria. Additionally, the release of sodA has been demonstrated to be related with MscL in *B. subtilis* [16]. We therefore explore the possibility of using these four non-classically secreted proteins as signals to export recombinant proteins.

**Fig. 1:**
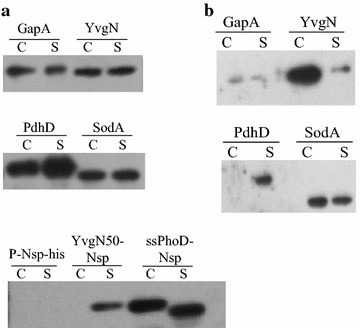
**a** Sub-cellular localization of Nsp fusions with non-classically secreted proteins and **b** sub-cellular localization of PhoA fusions with non-classically secreted proteins. Western blots of cellular (C) and supernatant (S) fractions from cells expressing non-classically secreted proteins fusions

Nsp from Baker’s yeast is an intrinsically disordered protein that fails to form 3-D structures under physiological conditions [24]. This characteristic minimises the influence of protein structure on secretion, so Nsp is seen as an ideal tool to study protein secretion [17]. Here, four non-classically secreted proteins (GapA, SodA, YvgN, PdhD) were fused to Nsp. Due to the rapid intracellular degradation of the unstructured Nsp, intracellularly expressed Nsp could not be detected in the cytoplasm or in the supernatant (Fig. 1a). Nsp that was exported with the signal peptide of PhoD was chosen as a control. The precursor Nsp with the signal peptide of PhoD was only detected in the cytoplasm, and the secreted Nsp led by the signal peptide of PhoD was only detected in the supernatant (Fig. 1a). This result suggests that the appearance of the fused proteins in the extracellular milieu is not due to cell lysis. The plasmids encoding GapA-Nsp, SodA-Nsp, YvgN-Nsp and PdhD-Nsp fusions were successfully transferred and expressed into *B. subtilis* WB800. All four fusions could be detected in the cytoplasm and supernatant by western blotting. In addition, the intracellular and extracellular sizes of these proteins were identical. These results show that there was no cleavage of the secreted proteins at either the N- or C-termini during the secretion process. The extracellular levels of these four fusion proteins were not less than the intracellular levels. Notably, the first 50 amino acids of YvgN were sufficient to direct the transport of Nsp to the medium, but Nsp could not be detected in the cytoplasm (Fig. 1a). All four non-classically secreted proteins were able to lead the secretion of the unstructured protein Nsp. Further research is needed to identify the secretion signals of these non-classically secreted proteins.

### Localisation of PhoA fusions to non-classically secreted proteins

Nsp can be exported to the culture medium with the aid of non-classically secreted proteins, but Nsp is unstructured and not suitable for quantitative assay. We therefore chose the frequently used reporter protein PhoA which is from *Escherichia coli* K12 as another model protein to study the possibility of using non-classically secreted proteins to lead the secretion of recombinant proteins. The correct folding of PhoA occurs only when it is secreted into the extracellular milieu. This location-specific folding property of this enzyme has led to its wide use as a reporter of protein localisation in prokaryotic cells [18]. Here, four non-classically secreted proteins were fused to PhoA. The relative activity of PhoA was measured in the supernatant (Table 1), and the expression of PhoA fusion proteins was checked by western blotting (Fig. 1b). All four fusions were detected in the cytoplasm by western blotting. Three of the four fusions (YvgN-PhoA, PdhD-PhoA and SodA-PhoA) were also detected extracellularly. SodA was the best performing non-classically secreted proteins and exported about 50 % PhoA to the culture medium. As shown in Fig. 1b and Table 1, the amount of fusion proteins in the supernatants generally correlated with the corresponding PhoA activities. SodA-PhoA fusion showed a PhoA activity of 36 % relative to the positive control. YvgN-PhoA showed about 17 % of PhoA activity compared to the control and GapA-PhoA had 8 % activity relative to the control. In contrast to these three fusions, PdhD-PhoA only resulted in background activity although it was present in the cytoplasm. These results show that PdhD cannot direct the export of PhoA. The other three proteins (SodA, YvgN and GapA) led the secretion of PhoA at different yield levels.

**Table 1:** Relative extracellular AP activity of fusions of PhoA with the native signal peptide

Name	Relative PhoA activity (%)^a^
YvgN-PhoA	17
SodA-PhoA	36
PdhD-PhoA	3
GapA-PhoA	8
pMA5ΔMCS	2

The results represent data from six independent experiments

^a^Extracellular AP activity of strains expressing PhoA fusions as percentage of activity of PhoA with the native signal peptide

### Localisation of BgaB fusions to non-classically secreted proteins

Thermostable β-galactosidase (BgaB) from *Geobacillus stearothermophilus* IAM11001 catalyses the hydrolysis of β-galactosides into monosaccharides and could be an ideal candidate for dairy processing [25], but it is difficult to obtain purified BgaB because this enzyme is cytoplasmic. Many efforts have been made to export BgaB to the extracellular medium, but it cannot be exported with the aid of typical Sec signal peptides [26]. In this study, four non-classically secreted proteins were tested for use as signals to lead the secretion of BgaB. The plasmids encoding GapA-BgaB, SodA-BgaB, YvgN-BgaB and PdhD-BgaB fusions were successfully transformed into the expression strain *B. subtilis* WB800. As a negative control, the plasmid p-ssNprE-BgaB-his was used to transfer WB800. The intracellular BgaB activities of the four strains (except the strain containing GapA-BgaB) were similar (Table 2). With the aid of YvgN, extracellular BgaB activity reached about 1.2 U/ml, and about 50 % BgaB was exported into the growth medium. The other three strains displayed less than 0.1 U/mL extracellular BgaB activity. No extracellular BgaB activity was detected in the negative control (Table 2). These results show that YvgN can lead the secretion of BgaB.

**Table 2:** The BgaB activities of BgaB fusion proteins in the cellular and the supernatant

Name	Intracellular BgaB activities (U/ml)	Extracellular BgaB activities (U/ml)
pMA5	0.002	–
GapA-BgaB	0.006	–
SodA-BgaB	2.2	0.07
YceD-BgaB	2.5	0.09
YvgN-BgaB	2.9	1.2
ssNprE-BgaB	2.0	0.06

The results represent data from six independent experiments

– Not detected

## Discussion

During the past two decades, numerous efforts have been made to explore the potential of *B. subtilis* as an expression cell factory for the secretion of recombinant proteins [3, 6, 9]. In general, exported proteins are synthesised as precursors with a cleavable signal peptide. The signal peptide is needed to direct the proteins to the export pathway and distinguishes the exported proteins from cytoplasmic proteins. Of the identified four distinct secretory pathways of *B. subtilis*, the largest number of exported proteins is transported via the major Sec pathway. Only a few specific substrates are exported via the three other routes (the Tat pathway, the ATP-binding cassette (ABC) transporter pathway, or the pseudopilin export pathway) [27]. The overwhelming majority of recombination proteins are designed to be secreted to the extracellular medium via the major Sec pathway. A number of homologous proteins have been successfully secreted at high levels from *B. subtilis*, but heterologous protein secretion is usually very inefficient, and cytoplasmic proteins are usually not translocated across the cell membrane with the aid of signal peptides [3, 9, 10]. These factors limit the use of *B. subtilis* as a cell factory and highlight the importance of exploring new transport routes for secreted heterologous proteins with the aim of large-scale commercial exploitation of *B. subtilis*.

More than 20 years ago, it was reported that interleukin 1 and galectin-1 can be exported from cells in the absence of any identifiable signal peptides. Since then, exported proteins without signal peptides have been identified by several researchers in various microorganisms [7, 11–15, 19–22], and the list of proteins known to be released without signal peptides is steadily growing. These proteins are generally present in the late stationary phase and are highly abundant in cytoplasmic proteomes, so the appearance of these proteins has attributed to cell lysis. However, recent reports have indicated that the secretion of these proteins is not due to cell lysis but is rather a general phenomenon [13]. In *B. subtilis*, the large conductance mechanosensitive channel protein MscL enables the specific release of cytoplasmic proteins during hypo-osmotic shock [16]. The mechanisms responsible for the secretion of these cytoplasmic proteins are becoming clearer, but no studies have attempted to utilise these pathways to export recombinant proteins into the culture medium.

In this study, the export of the three reporter proteins Nsp, PhoA and BgaB using four typical non-classically secreted proteins was tested. All four non-classically secreted proteins were able to direct the export of Nsp; about 50 % Nsp was transported to the extracellular milieu. This result suggests that Nsp meets the requirement of the substrate of the unknown translocation mechanism. However, Nsp is degraded easily so it is not suitable for quantitative analysis. PhoA was employed as another reporter protein. The activity of PhoA is location specific. Only when secreted into the extracellular milieu does PhoA display activity. When four non-classically secreted proteins were fused to PhoA, three (SodA, YvgN and GapA) were able to lead the secretion of PhoA, and the yield of PhoA differed according to the fusion protein. SodA-PhoA fusion showed the highest yield, giving a PhoA activity of 36 % relative to the positive control. GapA-PhoA detected in the supernatant had only 8 % activity relative to the control. Using the four non-classically secreted proteins as secretion signals, the yield of PhoA in the supernatant was low and the secretion efficiency was very low. However, these results further demonstrate that these non-classically secreted proteins are transported to the extracellular milieu and are not released by cell lysis. As secretion signals, they can lead PhoA to the exterior of the cell. These results also indicate that in addition to the secretion signals, the fusion proteins play an important role in protein translocation. Next, we attempted to use the four non-classically secreted proteins as signals to export BgaB, which cannot be secreted with the aid of typical signal peptides. YvgN was able to direct BgaB to the exterior of the cells, but the other three failed to do so. Although the secretion efficiency was not very high, the result shows that these proteins can lead recombinant proteins to the exterior of the cell, even if the recombinant protein is a cytosolic protein that cannot be export by the classical secretion pathway. The non-classical protein secretion pathway can be exploited as a novel secretion pathway for recombinant proteins, and is an excellent complement to the classical secretion pathway.

Although the non-classical secretion pathway can be used to secrete recombinant proteins, many aspects of this pathway require further study. First, more research is needed to explore the export mechanisms of these proteins or the signals that trigger their secretion. The size of non-classical protein is unlikely to influence the secretion of these reporter proteins. YvgN (about 30 kDa) can lead the secretion of Nsp (about 60 kDa), PhoA (about 47 kDa) and BgaB (about 78 kDa). SodA (about 22 kDa) and GapA (about 36 kDa) can direct the secretion of Nsp and PhoA. However, only PdhD (about 50 kDa) can guide the secretion of BgaB. It suggests that there is no correlation between the size of non-classical protein and the secretion of recombinant proteins. A recent publication identified a hydrophobic EM domain (residue 110–118) of enolase that is crucial for its secretion, and concluded that the structural EM element is important for enolase secretion [13]. Another study indicated that the covalent binding of the substrate/product 2-PG to a lysyl residue is important for the export of bacterial enolases into the medium [12]. These findings suggest that such regions could contain other secretion signals or be important for conformation suitable for secretion, but the secretion signals are unknown, and the question of how the secretory system distinguishes between its substrate and other cytoplasmic proteins remains. Second, although the three reporter proteins can be transported to the extracellular milieu with the aid of non-classically secreted proteins, the yield is very low. The maximum yield of PhoA led by non-classically secreted proteins is equal to only 36 % of PhoA led by the native signal peptide, and only about 50 % Nsp and BgaB are exported to the extracellular milieu with the aid of non-classically secreted proteins. As the yield and efficiency are both low, further studies are needed to improve protein production. Third, not all non-classically secreted proteins can direct the secretion of the given recombinant proteins. Only one of the four non-classically secreted proteins was able to lead the secretion of BgaB (about 78 kDa), but all of them were able to lead the secretion of Nsp (about 60 kDa). These findings show that the recombinant protein itself plays an important role in protein secretion when using the non-classical protein secretion pathway. Because PhoA (about 47 kDa) cannot be secreted by the aid of GapA, the size of reporter proteins could not be the main factors that influence their secretion. More research is needed to explore the favoured substrates in the non-classical protein secretion pathway and suitable non-classically secreted proteins for the desired target proteins. This study was the first to try to use non-classically secreted proteins as signals to export recombinant proteins to the culture medium. More studies are required to further explore this pathway for theoretical research and industrial applications.

## Conclusions

Secretory expression of cytoplasmic proteins into growth media has significant advantages. Due to the lack of an outer membrane, *B. subtilis* that exports recombinant proteins into the extracellular medium more easily has been explored as an expression cell factory for the secretion of recombinant proteins in the last two decades. However, the inefficiency of using the classical secretion pathway for the secretion of recombinant proteins limits its use on a wide scale. In this study, we successfully used four typical non-classically secreted proteins as signals to export three recombinant model proteins to the culture medium in *B. subtilis*. One of model proteins is the thermostable reporter protein β-galactosidase, which cannot be secreted using the classical secretion pathway. These results show that non-classically secreted proteins can lead recombinant proteins into the culture medium, and non-classical protein secretion pathways can be exploited as a novel secretion pathway for recombinant proteins in *B. subtilis*. More important, the non-classical protein secretion pathway is an excellent complement to the classical secretion pathway. This makes *B. subtilis* as an ideal cell factory for the secretory expression of recombinant proteins.

## Methods

### Bacterial strains, plasmids, and growth conditions

The plasmids and bacterial strains used are listed in Table 3. *Escherichia coli* DH5α was used as a host for cloning and plasmid manipulation. *Bacillus subtilis* WB800 [28] served as the protein expression host. *E. coli* and *B. subtilis* were grown in lysogeny broth (LB) at 37 °C supplemented with 100 μg/ml of ampicillin and 50 μg/ml of kanamycin, respectively.

**Table 3:** Strains and plasmids used in this study

Strain or plasmid	Genotype or characteristics	Source or reference
Strains		
*E. coli* DH5α	F^−^ *Ф80dlacZΔ (lacZYA*-*argF) U169 recA1 endA1 hsdR 17 (r*_*k*_^−^ *m*_*k*_^+^ *) supE44 λ* ^−^ *thi*-*1 gyrA relA1*	Lab. strain
*B. subtilis* 168	*trpC2*	Lab. strain
*B. subtilis* WB800	*nprE aprE epr bpr mpr::ble nprB::bsr vpr wprA::hyg*	[28]
Plasmids		
pMA5ΔMCS	An intermediate plasmid excised from the original cloning site of pMA5	[17]
p-Nsp-his	Encodes the unstructured protein Nsp	[17]
p-ssPhoD-Nsp-his	Encodes Nsp fused with signal peptide of PhoD	[17]
p-ssNprE-BgaB-his	Encodes BgaB fused with the signal peptide of NprE	This study
p-NCP-Nsp-his^a^	Encodes four non-classically secreted protein-Nsp fusions	This study
p-NCP-PhoA-his	Encodes four non-classically secreted protein-PhoA fusions	This study
p-YvgN50-Nsp-his	Encodes the first 50 amino acids of YvgN fused with Nsp	This study
p-PhoA-his	Encodes intact *Escherichia coli* PhoA	This study
p-NCP-BgaB-his	Encodes four non-classically secreted protein-BgaB fusions	This study

The four non-classically secreted proteins are GapA, PdhD, SodA and YvgN

^a^NCP represents the non-classically secreted proteins

### Plasmid construction

The primers used are listed in Additional file 1: Table S1. The plasmids used are listed in Table 3. The plasmids pMA5ΔMCS, p-Nsp-his, p-ssPhoD-Nsp-his, and p-ssNprE-BgaB-his were constructed during previous research [17]. To construct p-PhoA-his, a fragment of the *phoA* gene was amplified using *E. coli* DNA as the template with primers 9 and 11, digested with NdeI and BamHI, then cloned into the NdeI and BamHI sites of pMA5ΔMCS. The encoding sequences of four non-classically secreted proteins (PdhD, SodA, GapA and YvgN) were amplified from the genomic DNA of *B. subtilis* with primers 1–8. The PCR fragments were digested with NdeI and EcoRI (NdeI and MfeI for the *pdhD* gene), and ligated into NdeI/EcoRI-digested p-ssNprE-Nsp-his and p-ssNprE-BgaB-his, to give p-NCP-Nsp-his and p-NCP-BgaB-his, respectively. To make p-NCP-PhoA-his, *phoA* was first amplified using gene splicing by overlap extension PCR with primers 12–15 to remove EcoRI sites from the *phoA* gene, then amplified with primers 10–11 and cloned into the EcoRI and BamHI sites of p-NCP-Nsp-his.

### Transformation of DNA

Competent cells of *E. coli* DH5α were prepared and transformed as described elsewhere [29]. Competent cells of *B. subtilis* WB800 were prepared and transformed with plasmid DNA as described elsewhere [30]. The transformants were selected on LB agar plates containing 50 μg/ml of kanamycin.

### Gel electrophoresis and western blotting

Cells were grown at 37 °C on 5 ml of LB medium with 50 μg/ml of kanamycin or 100 μg/ml of ampicillin with shaking for 18 h. Cell growth was monitored by measuring the optical density at 600 nm (OD_600_). SDS–polyacrylamide gel electrophoresis (SDS-PAGE) was performed under reducing conditions using a 5 % stacking gel and a 12 % separating gel. Cells were ruptured by sonication, and centrifuged to precipitate and remove large debris. The culture supernatants and cells for SDS-PAGE were concentrated 10 times by 10 % trichloroacetic acid (TCA) and centrifugation. The samples in the SDS loading buffer were incubated for 10 min at 100 °C. Equal volumes of the secreted protein fraction and whole cells were applied to the gel and separated by SDS-PAGE. The proteins were then stained with Coomassie Blue. The protein samples for the immunoblot analysis were diluted four times, and after electrophoresis were transferred to polyvinylidene difluoride (PVDF) membranes, analysed with His tag antibodies and goat anti-mouse IgG, and exposed to X-ray film.

### Enzymatic assays

PhoA assays were performed as described previously [18]. After centrifugation, 100 μl of the supernatant was immediately diluted to 0.5 ml in Tris–HCl (pH 8.0), mixed with 0.5 ml of 0.5 % pNPP, and incubated at 37 °C for 10 min. The reaction was stopped by the addition of 0.5 ml of 2 N NaOH, and the OD_410_ was read. Enzyme activity was normalised to the same OD_600_. The relative PhoA activity values for the fusions were normalised relative to the PhoA activity in *B. subtilis* WB800 containing the p-PhoA-his. The activity of β-galactosidase BgaB was determined as described previously [25]. One unit of enzyme activity was defined as the amount of enzyme hydrolysing 1 μmol of substrate (ONPG) per minute.

## Additional file

10.1186/s12934-015-0374-6 Primers used for the PCR amplification.

## References

[1] Choi JH, Lee SY (2004). Secretory and extracellular production of recombinant proteins using *Escherichia coli*. Appl Microbiol Biotechnol.

[2] Low KO, Mahadi NM, Illias RM (2013). Optimisation of signal peptide for recombinant protein secretion in bacterial hosts. Appl Microbiol Biotechnol.

[3] van Dijl JM, Hecker M (2013). *Bacillus subtilis*: from soil bacterium to super-secreting cell factory. Microb Cell Fact.

[4] Demain AL, Vaishnav P (2009). Production of recombinant proteins by microbes and higher organisms. Biotechnol Adv.

[5] Guan C, Cui W, Cheng J, Zhou L, Guo J, Hu X, Xiao G, Zhou Z (2015). Construction and development of an auto-regulatory gene expression system in *Bacillus subtilis*. Microb Cell Fact.

[6] Wong SL (1995). Advances in the use of *Bacillus subtilis* for the expression and secretion of heterologous proteins. Curr Opin Biotechnol.

[7] Antelmann H, van Dijl JM, Bron S, Hecker M, Humphery-Smith I, Hecker M (2006). Microbial proteomics: functional biology of whole organisms. Proteomic survey through secretome of *Bacillus subtilis*.

[8] Monteferrante CG, MacKichan C, Marchadier E, Prejean MV, Carballido-López R, van Dijl JM (2013). Mapping the twin-arginine protein translocation network of *Bacillus subtilis*. Proteomics.

[9] Simonen M, Palva I (1993). Protein secretion in Bacillus species. Microbiol Rev.

[10] Li W, Zhou X, Lu P (2004). Bottlenecks in the expression and secretion of heterologous proteins in *Bacillus subtilis*. Res Microbiol.

[11] Harth G, Horwitz MA (1997). Expression and efficient export of enzymatically active *Mycobacterium tuberculosis* glutamine synthetase in *Mycobacterium smegmatis* and evidence that the information for export is contained within the protein. J Biol Chem.

[12] Boël G, Pichereau V, Mijakovic I, Mazé A, Poncet S, Gillet S, Giard JC, Hartke A, Auffray Y, Deutscher J (2004). Is 2-Phosphoglycerate-dependent automodification of bacterial enolases implicated in their export?. J Mol Biol.

[13] Yang CK, Ewis HE, Zhang X, Lu CD, Hu HJ, Pan Y, Abdelal AT, Tai PC (2011). Nonclassical protein secretion by *Bacillus subtilis* in the stationary phase is not due to cell lysis. J Bacteriol.

[14] Bendtsen ID, Wooldridge KG, Wooldridge K (2009). Non-classical secretion. Bacterial secreted proteins: secretion mechanisms and role in pathogenesis.

[15] Wang G, Chen H, Xia Y, Cui J, Gu Z, Song Y, Chen YQ, Zhang H, Chen W (2013). How are the non-classically secreted bacterial proteins released into the extracellular milieu?. Curr Microbiol.

[16] Kouwen TR, Antelmann H, van der Ploeg R, Denham EL, Hecker M, van Dijl JM (2009). MscL of *Bacillus subtilis* prevents selective release of cytoplasmic proteins in a hypotonic environment. Proteomics.

[17] Wang G, Chen H, Zhang H, Song Y, Chen W (2013). The Secretion of an intrinsically disordered protein with different secretion signals in *Bacillus subtilis*. Curr Microbiol.

[18] Payne MS, Jackson EN (1991). Use of alkaline phospatase fusions to study protein secretion in *Bacillus subtilis*. J Bacteriol.

[19] Voigt B, Schweder T, Sibbald MJ, Albrecht D, Ehrenreich A, Bernhardt J, Feesche J, Maurer KH, Gottschalk G, van Dijl JM, Hecker M (2006). The extracellular proteome of *Bacillus licheniformis* grown in different media and under different nutrient starvation conditions. Proteomics.

[20] Trost M, Wehmhöner D, Kärst U, Dieterich G, Wehland J, Jänsch L (2005). Comparative proteome analysis of secretory proteins from pathogenic and nonpathogenic *Listeria* species. Proteomics.

[21] Pasztor L, Ziebandt AK, Nega M, Schlag M, Haase S, Franz-Wachtel M (2010). Staphylococcal major autolysin (Atl) is involved in excretion of cytoplasmic proteins. J Biol Chem.

[22] Ziebandt AK, Becher D, Ohlsen K, Hacker J, Hecker M, Engelmann S (2004). The influence of agr and sigma B in growth phase dependent regulation of virulence factors in *Staphylococcus aureus*. Proteomics.

[23] Katakura Y, Sano R, Hashimoto T, Ninomiya K, Shioya S (2010). Lactic acid bacteria display on the cell surface cytosolic proteins that recognize yeast mannan. Appl Microbiol Biotechnol.

[24] Yamada J, Phillips JL, Patel S, Goldfien G, Calestagne-Morelli A, Huang H, Reza R, Acheson J, Krishnan VV, Newsam S, Gopinathan A, Lau EY, Colvin ME, Uversky VN, Rexach MF (2010). A bimodal distribution of two distinct categories of intrinsically disordered structures with separate functions in FG nucleoporins. Mol Cell Proteomics.

[25] Dong YN, Liu XM, Chen HQ, Xia Y, Zhang HP, Zhang H, Chen W (2011). Enhancement of the hydrolysis activity of β-galactosidase from *Geobacillus stearothermophilus* by saturation mutagenesis. J Dairy Sci.

[26] Xia Y, Zhao J, Chen H, Liu X, Wang Y, Tian F, Zhang HP, Zhang H, Chen W (2010). Extracellular secretion in *Bacillus subtilis* of a cytoplasmic thermostable β-galactosidase from *Geobacillus stearothermophilus*. J Dairy Sci.

[27] Tjalsma H, Antelmann H, Jongbloed JD, Braun PG, Darmon E, Dorenbos R, Dubois JY, Westers H, Zanen G, Quax WJ, Kuipers OP, Bron S, Hecker M, van Dijl JM (2004). Proteomics of protein secretion by *Bacillus subtilis*: separating the “secrets” of the secretome. Microbiol Mol Biol Rev.

[28] Wu SC, Yeung JC, Duan Y, Ye R, Sazrka SJ, Habibi HR, Wong SL (2002). Functional production and characterization of a fibrin-specific single-chain antibody fragment from *Bacillus subtilis*: effects of molecular chaperones and a wall-bound protease on antibody fragment production. Appl Environ Microbiol.

[29] Cohen SN, Chang AC, Hsu L (1972). Nonchromosomal antibiotic resistance in bacteria: genetic transformation of Escherichia coli by R-factor DNA. Proc Natl Acad Sci USA.

[30] Anagnostopoulos C, Spizizen J (1961). Requirements for transformation in *Bacillus subtilis*. J Bacteriol.

