# Supplementary material for: A new potential secretion pathway for recombinant proteins in Bacillus subtilis
Source: Microb Cell Fact. 2015 Nov 10;14:179. doi: 10.1186/s12934-015-0374-6 (PMC4641360; doi:10.1186/s12934-015-0374-6)
Supplement: Supplementary file 1 — 10.1186/s12934-015-0374-6 Primers used for the PCR amplification. [file 12934_2015_374_MOESM1_ESM.doc]

Table S1. Primers used for the PCR amplification

| Number | Name | Sequence (5'→3') |
| --- | --- | --- |
| 1 | GapANdeIF | ATCAATTGCATATGGCAGTAAAAGTCGGTATTAAC |
| 2 | PdhDNdeIF | GCGTCAATTGCATATGGTAGTAGGAGATTTCCCT |
| 3 | SodANdeIF | GCGTGAATTCCATATGGCTTACGAACTTCCAGAATT |
| 4 | YvgNNdeIF | GCGTGAATTCCATATGCCAACAAGTTTAAAAGATAC |
| 5 | GapAEcoRIR | CAATGAATTCGGATCCAAGACCTTTTTTTGCGATGT |
| 6 | PdhDMfeIR | GCATCAATTGGGATCCTTTTACGATGTGAATCGGACT |
| 7 | SodAEcoRIR | GCATGAATTCGGATCCTTTTGCTTCGCTGTATAGACG |
| 8 | YvgNEcoRIR | GCATGAATTCGGATCCAAACAGAAGCTCATCAGGAT |
| 9 | PhoANdeIF | GCCGGCATATGAAACAAAGCACTATTGCACTGG |
| 10 | PhoAEcoRIF | GCTACGAATTCCGGACACCAGAAATGCCTGTTC |
| 11 | PhoABamHIR | CTGGATCCTTTCAGCCCCAGAGCGGCTTTCATG |
| 12 | phoA794gaiF | GCGATGCTGCCTCACTCAACTCGGTGACGGAAGCGAATC |
| 13 | phoA794gaiR | GATTCGCTTCCGTCACCGAGTTGAGTGAGGCAGCATCGC |
| 14 | phoA1125gaiF | CGTACAACGGGCGCTGGAGTTTGCTAAAAAG |
| 15 | phoA1125gaiR | CTTTTTAGCAAACTCCAGCGCCCGTTGTACG |
